# SYNbiotics Easing Renal failure by improving Gut microbiologY (SYNERGY): a protocol of placebo-controlled randomised cross-over trial

**DOI:** 10.1186/1471-2369-15-106

**Published:** 2014-07-04

**Authors:** Megan Rossi, David W Johnson, Mark Morrison, Elaine Pascoe, Jeff S Coombes, Josephine M Forbes, Brett C McWhinney, Jacobus PJ Ungerer, Goce Dimeski, Katrina L Campbell

**Affiliations:** 1 School of Medicine, University of Queensland, Brisbane, Australia; 2 Human Movement Studies, University of Queensland, Brisbane, Australia; 3 Diamantina Institute, University of Queensland, Brisbane, Australia; 4 Translational Research Institute, Brisbane, Australia; 5 Department of Nephrology, Princess Alexandra Hospital, Brisbane, Australia; 6 Chemical Pathology, Princess Alexandra Hospital, Brisbane, Australia; 7 Mater Medical Research Institute, Brisbane, Australia; 8 Department of Chemical Pathology, Pathology Queensland, Brisbane, Australia

**Keywords:** Prebiotics, Probiotics, Synbiotics, Chronic kidney disease, Gut microbiota, Indoxyl sulphate, P-cresyl sulphate, Endotoxins

## Abstract

**Background:**

Emerging evidence suggests modulating the microbiota in the large bowel of patients with chronic kidney disease (CKD) through pre- and/probiotic supplementation may inhibit the development of key nephrovascular toxins. To date, quality intervention trials investigating this novel treatment in CKD are lacking. The aim of SYNERGY is to assess the effectiveness of synbiotics (co-administration of pre- and probiotics) as a potential treatment targeting the synthesis of uremic toxins, specifically, indoxyl sulphate (IS) and p-cresyl sulphate (PCS).

**Methods/design:**

Thirty-seven patients with moderate to severe CKD (Stage IV and V, pre-dialysis) will be recruited to a double-blind, placebo-controlled, randomised cross-over trial. Patients will be provided with synbiotic therapy or placebo for 6 weeks, with a 4 week washout before cross-over. The primary outcome is serum IS, total and free (unbound) concentrations, measured using ultra-performance liquid chromatography. Secondary outcomes include serum PCS, total and free (unbound) concentrations; cardiovascular risk, measured by serum lipopolysaccharides, serum trimethylamine-N-oxide (TMAO) and inflammation and oxidative stress markers; kidney damage, measured by 24 hour proteinuria and albuminuria, estimated glomerular filtration rate and renal tubule damage (urinary kidney injury molecule-1); patients’ self assessed quality of life; and gastrointestinal symptoms. In addition, the effects on the community structure of the stool microbiota will be explored in a subset of patients to validate the mechanistic rationale underpinning the synbiotic therapy.

**Discussion:**

IS and PCS are two novel uremic toxins implicated in both cardiovascular disease (CVD) and progression of CKD. Preliminary studies indicate that synbiotic therapy maybe a promising strategy when considering a targeted, tolerable and cost-efficient therapy for lowering serum IS and PCS concentrations. This trial will provide high quality ‘proof-of-concept’ data to elucidate both the efficacy of synbiotic therapy for lowering the toxins and whether reductions in serum IS and PCS translate into clinical benefits. Considering the potential of pre- and probiotics to not only shift toxin levels, but to also impede CVD and CKD progression, SYNERGY will provide vital insight into the effectiveness of this innocuous nutritional therapy.

**Trial Registration:**

Universal Trial Number: U1111-1142-4363. Australian New Zealand Clinical Trials Registry Number: ACTRN12613000493741, date registered: 2^nd^ May 2013.

## Background

In recent years, an appreciation for the role of the gut microbiota in health and disease has gained momentum, with microbial modulating therapies emerging in mainstream medicine [1]. Within the discipline of Nephrology, the evidence supporting the role of the kidney-gut axis in uremia is building [2]. In fact, it is now clear that the dysbiotic gut microbiotia observed in chronic kidney disease (CKD) [3] produce key nephrovascular toxins, indoxyl sulphate (IS) and p-cresyl sulphate (PCS) [4].

There is convincing evidence demonstrating dose-dependent nephro- and cardiovascular toxicities of IS and PCS in both *in vitro* and animal studies [5-7]. Further, in the past decade there has been a surge of observational studies describing these toxins and their associations with increased cardiovascular disease (CVD), kidney disease progression and all-cause mortality in the CKD population [8-12]. This growing body of observational literature warrants the need for more conclusive findings from intervention studies to elucidate whether there is a causal role of IS and PCS in the cardiorenal milieu, or whether they are in fact biomarkers *in vivo*.

A number of therapeutic opportunities for targeting IS and PCS have been proposed, including inhibition of colonic bacterial biosynthesis (protein restriction and microbial modulating therapies), suppression of absorption (oral adsorbents), augmentation of clearance (enhanced dialysis) and modulation of cellular pathways (organic anion transporters and antioxidants) [13]. Many of these therapies remain limited to experimental studies, have unfavourable side effects or a high cost burden preventing their translation to clinical research. In particular, oral adsorbents have been extensively studied, with promising improvements in both cardiovascular risk [14] and kidney function [15] following reductions in serum IS. Nonetheless, a recent Phase III trial (n = 2035), Evaluating Prevention of Progression In Chronic Kidney Disease (EPPIC), demonstrated no difference between placebo and intervention (AST-120) in CKD progression. Interestingly, post-hoc analysis suggested poor compliance may have contributed to the negative outcome [16]. Microbial modulating therapies, in the form of pre- and probiotcs, however, present themselves as a promising treatment given their low cost and innocuous nature. The potential benefit and definition of pre- and probiotics are outlined in Table 1[17,18]. These therapies have been summarised by a systematic review and meta analysis, which suggested an overall benefit for reducing the production of IS and PCS by first altering the microbiota community in the large bowel and thereby the metabolic activity and formation of these metabolites within the colon [19]. However, this review highlighted the body of evidence was weak due to poor study design, suboptimal methodologic quality, significant trial heterogeneity, and lack of scientific rationale for each study’s supplement selection (probiotic strains, prebiotic varieties and dosing). In addition, none of the trials evaluated in this review were undertaken in the pre-dialysis population where it may have the greatest therapeutic benefit for delaying CKD progression, nor did they investigate the effect of lowering the toxins on clinically relevant markers and outcomes.

**Table 1:** Mechanisms underpinning the scientific rationale for the selection of the synbiotic formulation targeting the production of indoxyl sulphate and p-cresyl sulphate

**Prebiotics ***“A selectively fermented ingredient that allows specific changes, both in the composition and/or activity in the gastrointestinal microflora that confers benefits upon host wellbeing and health” *[17]	Selectively support the colonization of probiotics
	Increase the carbohydrate:nitrogen ratio in the colon favouring beneficial saccharolytic vs. proteolytic fermentation
	Decrease colonic pH (through short chain fatty acid production) thereby impairing protein degradation with inactivation of pH sensitive proteases
	Increase the colonic transit time, thereby decreasing time for bacterial production and intestinal absorption of indoxyl sulphate and p-cresyl sulphate
	Enhance bacterial growth with increased uptake of the amino acids for bacterial biosynthesis and therefore less substrate for protein fermentation
**Probiotics***“Live microorganisms which when administered in adequate amounts confer a health benefit on the host” *[18]	Acid and bile resistance to ensure survival through the upper gastrointestinal tract
	Competitive exclusion of indoxyl sulphate and p-cresyl sulphate producing bacteria (through competition for essential nutrients and luminal and epithelial binding sites)
	Direct bacterial antagonism via inhibitory substance production (such as biosurfactants, hydrogen peroxide, and bacteriocins)
	Immunomodulation via immune cell activation resulting in indirect inhibition of pathogenic bacteria

For these reasons, high quality studies are needed to address 1) the efficacy of synbiotic therapy for reducing IS and PCS and 2) whether such reduction in toxin concentrations is associated with clinical benefit. Answers to these fundamental questions will ascertain the clinical applicability of synbiotic therapy in the CKD setting; and also provide new insight into how the microbiota may be redirected to promote better clinical management of CKD.

## Methods/design

### Study aim

The aim of the *SYNbiotics: Easing Renal failure by improving Gut microbiologY* (SYNERGY) study is to investigate the effects of synbiotics (co-administration of pre- and probiotics) as a potential treatment for reducing IS and PCS production in the CKD population. The primary hypothesis is that synbiotic supplementation in patients with Stage IV or V CKD (pre-dialysis) will be effective in reducing accumulation of total and free (unbound) concentrations of IS. The secondary hypotheses for SYNERGY include: a) reduction in total and free concentrations of PCS; b) reduction in cardiovascular risk- measured by reduced endotoxemia, trimethylamine-N-oxide (TMAO) and inflammatory and oxidative stress markers; c) reduction in kidney damage- measured by proteinuria and albuminuria and renal tubule damage (urinary kidney injury molecule-1 (Kim-1)); d) improvement in quality of life (QOL); and e) improvement in gastrointestinal (GI) symptoms.

### Study design

**Figure 1:**
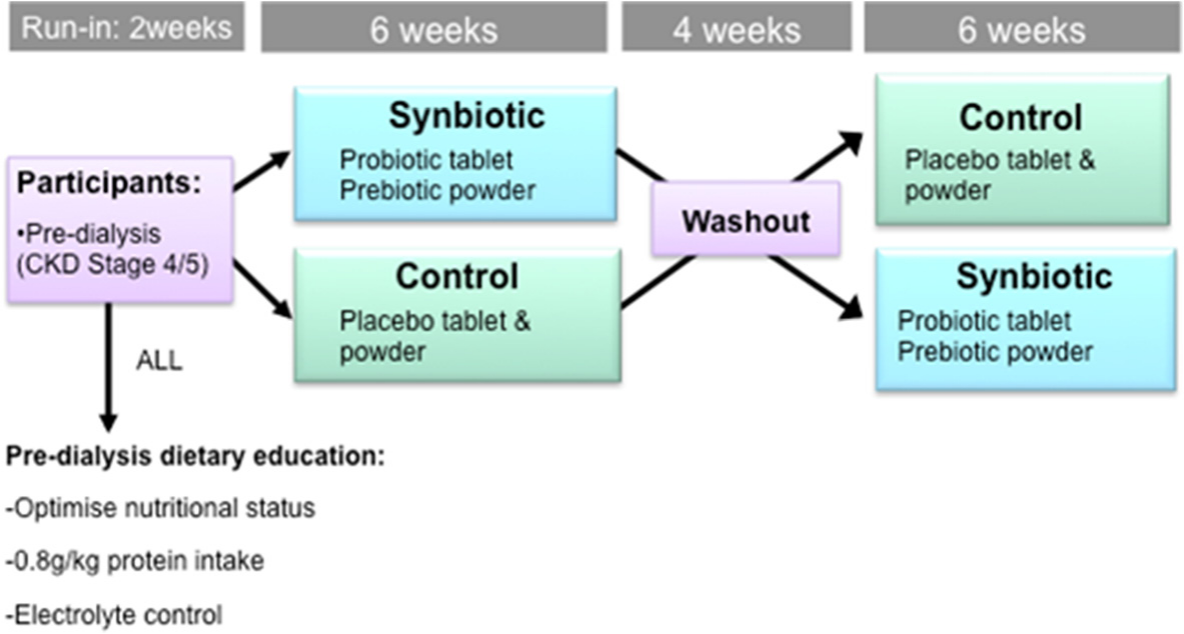
SYNERGY study schema.

SYNERGY is a single-centre, double-blind, placebo-controlled, randomised cross-over trial. Participants will undergo a 2 week run-in period, followed by randomisation to either synbiotic supplements or placebo for 6 weeks. Thereafter, participants will undergo a further 4 week washout period followed by crossover to the alternative intervention (Figure 1). The 4 week washout is considered sufficient for the pre-intervention microbiota to re-establish [19].

### Ethical considerations

Ethical approval has been granted through Metro South Human Research Ethics Committee and the University of Queensland Human Research Ethics Committee. Further, Therapeutic Goods Administration has approved the synbiotic supplements under the Clinical Trials Notification Scheme.

### Target population

The trial will recruit Stage IV-V non-dialyzed CKD patients. Inclusion criteria will include patients under the care of a Nephrologist at the Princess Alexandra Hospital with an estimated glomerular filtration rate (eGFR) between 10-30 ml/min/1.73 m^2^, aged ≥18 years and able to provide informed consent. Patients will be excluded if they meet any of the following criteria: previous renal transplant; receiving/or have received radiation to the bowel or large bowel resection; consumed pre- or probiotics or antibiotic therapy within 1 month of study commencement; medically diagnosed irritable bowel syndrome, Crohn’s disease or ulcerative colitis; non-English speaking; likely to receive a transplant or progress to dialysis within 6 months, as determined by treating physician; severely malnourished (Subjective Global Assessment: C); or having had a clinically significant change to their immunosuppressant dose within 6 months (determined by the medical team). The latter criterion is to mitigate any risk associated with severely immunocompromised patients, although increased risk even in the critically ill is thought to be minimal [20].

### Dietary counselling

All participants will undergo face-to-face dietary education and counseling with a qualified dietitian in line with evidence-based guidelines [21], incorporating standard pre-dialysis education during the first week of run-in (Figure 1). Specifically, the goal of the intervention provided will be to target intake in line with current recommendations [22], including protein intake at 0.8 g/kg/day and to establish baseline dietary fibre intakes. In the week following, and throughout the intervention, patients will be encouraged to maintain stable protein and fibre intakes, with specific attention to maintaining the same sources of these nutrients (ie. animal vs. plant protein and soluble vs. insoluble fibre). Participants’ dietary intakes and adherence to a stable diet during the study will be assessed using a multi-method approach to optimize accuracy of dietary intake estimates while minimizing patient burden. An open-ended, structured diet history method will be used to establish usual intake at baseline and the end of each intervention period. This method is considered ideal for capturing usual intake over a predefined period of time. In order to limit recall bias, a self-administered diet history will be used based on a template completed prior to the interview [23], and verified by the same dietitian in a face-to-face interview. A 24 hr recall, according to the US Department of Agriculture multiple pass method [24], will be employed to assess the stability of dietary intake throughout the study. This less time-intensive dietary assessment method was chosen to limit participant burden. Both diet assessment methods will utilize food models to increase the accuracy of estimated portions, particularly protein and fibre sources. Dietary data will be entered into Food Works 7 (Xyris Software, version 7.0.2915) using the Australian Food, Supplement and Nutrient database (AUSNUT) 2007 (for key macro- and micronutrients) and NZ Foodfiles 2010 (to quantify soluble fibre and amino acids, not available in AUSNUT). In addition, dietary protein intakes will be verified according to the formula by Maroni et al. [25] using 24 hr urinary urea nitrogen and body weight [26].

### Randomisation

Computer-generated randomisation of participants to treatment order will be undertaken by an external statistical consultant. This process of allocation will conceal the randomisation order to researchers and participants. In addition, the supplements will be packed off-site with a generic label, supplement A or B, for the first and second intervention, respectively.

### Pre- and probiotic intervention

The underlying rationale for selecting the bacterial strains in the synbiotic formulation is the mechanistic inhibition of bacterial production of IS and PCS (detailed in Table 1) [27]. Importantly, the bacterial strains selected should have limited, if at all, enzymatic capacity to produce IS and/or PCS and ideally displace bacteria that do. On the basis of these criteria, strains from the Lactobacillus and Bifidobacteria genera present themselves as suitable candidates for the purpose of this trial [28-30].

A strain from the *Streptococcus thermophilus* species is also considered an important component in the synbiotic formulation due to its high urease capacity. In fact, a previous study in the CKD population reported a significant decrease in blood urea nitrogen (BUN) with the inclusion of this species in the probiotic formula [31]. Hence, the probiotics in this study will contain strains from the Lactobacillus, Bifidobacteria and Streptococcus genera.

The prebiotic component will include high molecular weight inulin (inulin HP), fructo-oligosaccarides (FOS) and galacto-oligosaccarides (GOS), based on a number of mechanistic benefits described in Table 1[32,33]. In addition, the literature suggests the production of IS and PCS occurs in both the proximal and distal sections of the colon. Therefore, in order to target and diminish the colonic production of these toxins, a prebiotic product that facilitates fermentation throughout the entire colon is ideal. FOS and GOS both have small degrees of polymerisation (DP) (DP < 10) and therefore are completely fermented in the proximal part of the colon. The addition of inulin HP with a DP ranging from 10–60 may assist the extension of the fermentation to the distal part of the colon [34].

In order to ensure the validity of the formulation, only fibre varieties that have gone through the rigorous process of attaining prebiotic status, as defined by Gibson et al. will be included [35].

### Supplement dosing and duration

The synbiotic intervention will be a daily dose of 15 grams of prebiotics, including a combination of 3 different types of fibres, and the probiotic component will include 90 billion colony-forming units (CFU) from 9 different strains across the Lactobacillus, Bifidobacteria and Streptococcus genus. There is no consensus as yet on adequate dosing or duration of synbiotics due to the diversity of GI survival rates of probiotic strains and the different characteristics of prebiotic varieties, such as their bifidogenic capacities [33]. Nevertheless, the available evidence suggests that there maybe be a threshold dose and duration required to see a benefit for lowering IS and PCS production [19]. The dosing of pre- and probiotics used in SYNERGY is based on previous successful trials [30,36,37]. However, in order to minimise the side effects reported in previous studies, such as flatulence and bloating [38], the synbiotics will commence at half dose for the first 3 weeks.

The durations of most intervention studies in this area have been ≤4 weeks, with one study demonstrating no benefit comparing 4, 8 and 12 week intervention periods [37]. The intervention period for SYNERGY is a 6 week duration to allow time for dose escalation, minimise symptom burden and establish whether there is a dose-dependent and sustained benefit of the therapy.

### Primary outcome

#### Serum indoxyl sulphate

Venous blood will be collected following an overnight fast at 7 time points throughout the study, see Table 2. Samples will be centrifuged at 3000 rpm for 10 mins before being stored at −80°C. Samples will be batched and sent for analysis of serum concentration of IS, total and free, using an ultra-performance liquid chromatography (UPLC) and fluorescence detection method. This recently validated method will allow for low limits of detection down to 0.1 μmol/L and has been described in detail by Pretorius et al. [39]. This method will also be followed for PCS.

**Table 2:** SYNERGY data collection schedule

		**Run-in Week −2**	**Intervention A & B**		
			**Baseline**	**Mid point**	**End point**
			**Week 0 & 11**	**Week 3 & 14**	**Week 6 & 17**
**Serum uremic toxins**	Indoxyl sulphate (free and total)	X	X	X	X
	P-cresyl sulphate (free and total)	X	X	X	X
**Cardiovascular risk**	Oxidative stress		X		X
	Inflammation		X		X
	Endotoxemia (lipopolysaccharide)		X		X
**Kidney damage**	24 hr albuminuria and protienuria		X		X
	Kidney injury molecule-1		X		X
	Estimated glomerular filtration rate	X	X		X
**Dietary**	Diet history interview	X			X
	24 hr recall		X	X	
	24 hr urinary urea nitrogen		X		X
**Quality of life**	Short Form-36				X
**Gastrointestinal symptoms**	Gastrointestinal symptom rating scale	X		X	X
**Gut microbiota**	Fecal sample (optional)		X		X
**Compliance**	Pill count			X	X
**Dose escalation**				X	

Participants will be provided with a standard evening meal preceding their overnight fast before each blood collection. This is a precautionary measure to minimise any potential residual influences of the macronutrient distribution of proximal meals on participants’ serum IS and PCS levels.

### Secondary outcomes

#### Endotoxemia

Plasma samples will be collected in endotoxin-free vials following each intervention and stored at −80°C. Quantification of lipopolysaccharides will be undertaken using a Limulus Amebocyte assay (Cambrex, Verviers, Belgium), as described previously [40].

#### Trimethylamine-N-oxide

TMAO and its precursor, trimethylamine (TMA), will be extracted from plasma with Acetonitrile-containing labelled internal standards. The supernatant will then be injected into a Waters UPLC system and separated under reverse phase conditions. The column eluent will be introduced into a Waters TQD Tandem Mass Spectrometer and specific Multiple Reaction Monitoring (MRM) will be carried out for each of the analytes.

#### Inflammation and oxidative stress

A panel of inflammatory markers, including interleukin-6 (IL-6) and tumor necrosis factor-α (TNFα), will be measured in serum samples before and after each intervention using electrochemiluminescence immunoassay techniques. Selection of these markers was based on their regulatory association with Nuclear Factor-kappaB (NF-kB) [41] and their association with IS and PCS in the CKD population [42].

In addition, markers of lipid oxidation (total F_2_ isoprostanes) and endogenous antioxidant activity (glutathione peroxidase (GPx)) will be measured to provide a comprehensive assessment of potential pathways involved in the toxicity of IS and PCS . These biomarkers will be measured in plasma samples using validated methods [43,44].

#### Kidney damage

SYNERGY will include kidney damage as a secondary outcome using 24 hr urine samples (gold standard) for measuring proteinuria and albuminuria [45]. Participants will be provided with bottles for collection and educated on correct collection procedures in accordance with standard protocol. Urinary total protein (pyrogallol red) and urinary albumin (turbidimetric method based on antibody-antigen complexes) will be measured in timed-samples (mg/24 hrs) and cross-checked against creatinine ratios (protein and albumin, respectively) on Beckman DxC800 general chemistry analyser (Beckman Coulter, Brea, CA, USA) [46].

Kidney function using the Chronic Kidney Disease Epidemiology Collaboration (CKD-EPI) formula will also be measured [22].

In addition, urinary Kim-1, a marker of kidney tubule damage, will be measured from midstream urine collections before and after each intervention [47]. Samples will be stored at −80°C, followed by analysis using a commercially available sandwich ELISA according to the manufacturer’s instructions (USCN Life Sciences, Wuhan, PRC). Significant changes in Kim-1 have been achieved in a 6 week dietary intervention study indicating the feasibility of Kim-1 as a sensitive marker in SYNERGY [48].

#### Quality of life

QOL will be assessed in SYNERGY by the validated Short Form-36 (SF-36), which has been used widely in renal populations [49,50].

#### Gastrointestinal symptoms

Monitoring GI symptoms in SYNERGY has two purposes, safety monitoring and hypothesis generation. The validated Gastrointestinal Symptom Rating Scale (GSRS) will be administered at baseline, mid-point (prior to dose escalation), and the end of each intervention [51]. The GSRS data will be presented as a total score and as dimension scores (reflux, pain, indigestion, constipation and diarrhoea) [52].

#### Safety and adherence

All serious adverse events will be reported to the ethics committee, whether deemed to be supplement related or not. In addition, medical officers will cross-check all blood and urine results during the study. Adherence to supplements will be measured by pill count and powder weight at both the mid point and end of each intervention.

### Exploratory outcome

#### Gut microbiota

A microbial analysis will be an opt-in component to SYNERGY. Fecal samples will be collected according to standard procedures and stored at −80°C prior to batch analysis pre and post each intervention. Microbial DNA extraction will be performed using published methods [53] and the abundance of key probiotic bacteria measured by species-specific qPCR methodologies. These specific bacteria as well as alpha- and beta-diversity measures will be derived from barcoded amplicon libraries of the V4 hypervariable region (517 F-803R) of the prokaryote *rrs* genes present in each sample, using protocols similar to those described by Caporaso et al. [54]. The libraries will be sequenced using the Illumina MiSeq platforms, and following demultiplexing and initial sequence quality control assessments, analyzed using the Quantitative Insights Into Microbial Ecology (QIIME) pipeline (qiime.sourceforge.net) as described by Caporaso et al. [55]. Enzymes relevant to the primary outcome of the study (namely tryptophanase and 4-hydroxyphenylacetate decarboxylase, which catalyze the production of IS and PCS, respectively) will also be measured. This is vital for proof of concept exploration and to provide insight for refinement of the synbiotic formulation.

### Statistical analysis

Preliminary data from a cohort of CKD Stage IV-V (pre-dialysis) patients recruited from the same Nephrology department were used for power calculations. The mean serum concentration of IS in this population was 14 μmol/L with a mean intra individual standard deviation of 4 μmol/L between 3 measurements over a 6 week period (unpublished data from McMahon and Campbell). With 1:1 randomisation, SYNERGY will require 24 participants to complete the study. This sample size is based on a 30% reduction in IS levels; alpha of 5% and power of 90%. Allowing for a 20% drop out and using the adjustment factor 1/1(1-υ)^2^, a total of 37 participants will be recruited. This magnitude of change is related to a significant improvement in kidney function [56]. Furthermore, a reduction in IS concentration of this size has been achieved in previous probiotic intervention studies and is therefore considered a realistic target [57].

The primary analysis of IS concentrations will be undertaken using an independent t-test with treatment sequence allocation as the independent variable and the difference between serum concentrations measured after the first and second treatment as the dependent variable [58]. Secondary analysis of the primary outcome will include mixed modelling to account for missing data and to determine whether there is a dose dependent effect of the synbiotics.

In addition to the widely used diversity metrics and analysis tools available via QIIME, permutational multivariate analysis of variance (PERMANOVA) will be conducted on the genus level operational taxonomic units (OUT) tables to test the relationships between the stool microbiome and the primary and secondary outcomes of the study. Constrained ordination methods, such as analysis with respect to instrumental variables, will also be carried out [59].

Previous studies in pre-dialysis populations suggest a high incidence of antibiotic prescription secondary to high comorbidity burden, susceptibility to urinary tract infections, and prophylactic use for surgical procedures. Because this is a proof-of-concept study and the use of antibiotics may confound the mechanisms underpinning the synbiotic therapy, sensitivity analyses will be undertaken including and excluding patients prescribed antibiotics during either intervention arm. In addition, further analysis will be undertaken to assess whether there is a treatment order effect. The null hypothesis will be rejected at the 0.05 level. The statistical analyses will be performed using Stata (version 12, 2012, Statacorp, College Station, TX).

## Discussion

This double-blind placebo-controlled randomised cross-over trial has been designed to provide evidence in order to better determine whether synbiotic therapy can reduce serum concentrations of IS and PCS and in turn improve clinical outcomes in CKD. In addition to overcoming suboptimal study design and methodological limitations of previous studies, SYNERGY aims to address the translation of the inflammatory and oxidative mechanisms that are thought to underpin the toxicities of IS and PCS contributing to the high prevalence of premature CVD observed in CKD [60]. More explicitly, *in vitro* studies suggest the pathogenic actions of IS and PCS stem from the induction of reactive oxygen species (ROS), which activate the NF-kB pathway, resulting in both oxidative stress and pro-inflammatory cytokine production [61,62]. Furthermore, treatment with antioxidants and NF-kB inhibitors dose-dependently inhibit the fibrotic and oxidative effects of IS and PCS [5,14,63]. SYNERGY will be one of the first intervention studies to investigate the translation of these mechanisms in the CKD population.

There are a number of other mechanistically plausible benefits of synbiotic therapy in CKD, independent of the effect on IS and PCS reduction, that will be explored in SYNERGY, specifically, endotoxemia, TMAO, QOL, GI symptoms and microbiota form and function.

Endotoxemia, a marker of impaired intestinal barrier function, has been identified across the full spectrum of CKD, with levels of circulating endotoxins increasing with declining kidney function [64]. Some of the factors contributing to this in CKD include the high prevalence of small bowel bacterial overgrowth [65], increased membrane permeability secondary to chronic low grade inflammation [66], and gut edema secondary to fluid overload and ischemic intestinal injury particularly in the dialysis population [64]. The potential benefit of probiotics on intestinal lining integrity has gained recognition recently, with studies in liver disease patients demonstrating a significant reduction in endotoxin levels following synbiotic therapy [67,68]. Some of the mechanisms proposed include enhanced epithelial barrier function via induction of mucin production, blocking of epithelial binding receptors, and strengthening of epithelial tight junctions through increased expression of proteins [69]. It is therefore conceivable that endotoxemia may improve following synbiotic therapy in CKD.

Like IS and PCS, TMAO is a derivative of colonic bacterial fermentation and has recently been identified as a proatherogenic risk factor [70]. Emerging research has demonstrated that TMAO not only promotes atherosclerosis in mice, but raised levels are associated with major adverse cardiac events in humans (n = 2595) [71]. Despite this link between TMAO and atherosclerosis only recently surfacing, increased levels of TMAO in end stage kidney disease and its amenability to gut microbial manipulation has been recognised for decades [72]. Therefore a decrease in serum TMAO is an attractive prospect following synbiotic therapy.

QOL in CKD patients is often compromised with levels of depression associated with declining kidney function [73]. There is a growing body of evidence substantiating the microbiota-gut-brain axis, linking alterations in the gut microbiota with depression [74]. Further, animal studies have demonstrated that both pre- [75] and probiotics [76] have psychotropic effects, suggesting improvements in QOL following synbiotic therapy is a credible hypothesis. In fact, this theory has been supported in a randomised controlled cross-over study where a cohort of 46 participants with CKD stage 3–4 were reported to have a significant improvement in their QOL following probiotic therapy [77].

GI symptoms are increased in CKD compared to the general population. Furthermore, an observational study comparing the CKD population with the general population highlighted significantly worse GI symptom across 5 GI domains (pain, indigestion, constipation, diarrhoea and eating dysfunction) using the GSRS tool [52]. Given the reported benefits of pre- and/or probiotics span across these 5 GI domains [78], GI symptom improvement presents itself as another promising outcome measure in SYNERGY.

The microbiota analysis in this study should help evaluate whether the probiotic strains *per se,* or other alterations in the microbiota of the large bowel, might contribute to these outcomes.

Lastly, the role of diet in serum IS and PCS concentrations is well documented, yet monitoring dietary intake has been a limitation of studies to date [79]. One of the key strengths of SYNERGY that will enhance the rigour of this trial is the multi-method approach to monitoring participants’ dietary intakes throughout the study.

In summary, considering the potential for synbiotics to not only shift toxin levels, but to also impact on CVD and CKD outcomes, there is a need for well designed, intervention studies to establish the effectiveness of this innocuous, low cost therapy. The SYNERGY study aims to provide proof-of-concept data to elucidate whether altering the microbiota in the CKD population is likely to be effective, tolerable and can impede the processes associated with CVD and CKD progression.

## Competing interests

The authors declare that they have no competing interests.

## Authors’ contributions

MR participated in trial design, development of statistical plan and drafting of the manuscript. KC and DJ participated in trial design, statistical plan review and editing of the manuscript. MM participated in trial design (specifically microbiota analyses) and editing of the manuscript. EP participated in development of the statistical plan and editing of the manuscript. JC participated in trial design (specifically inflammation and oxidative stress markers) and editing of the manuscript. JF participated in trial design (specially Kim-1 analysis) and editing of the manuscript. BM and JP participated in trial design (specifically uremic toxins and TMAO analysis) and editing of the manuscript. GD participated in trial design (general pathology analysis) and editing of the manuscript. All authors read and approved the final manuscript.

## Pre-publication history

The pre-publication history for this paper can be accessed here:

http://www.biomedcentral.com/1471-2369/15/106/prepub
